# Complementary genetic screens identify the E3 ubiquitin ligase CBLC, as a modifier of PARP inhibitor sensitivity

**DOI:** 10.18632/oncotarget.3628

**Published:** 2015-03-18

**Authors:** Jessica Frankum, Pavel Moudry, Rachel Brough, Zdenek Hodny, Alan Ashworth, Jiri Bartek, Christopher J. Lord

**Affiliations:** ^1^ The CRUK Gene Function Laboratory and Breakthrough Breast Cancer Research Centre, The Institute of Cancer Research, London, UK; ^2^ Danish Cancer Society Research Center, Strandboulevarden, Copenhagen, Denmark; ^3^ Institute of Molecular Genetics, Academy of Sciences of the Czech Republic, Videnska, Czech Republic

**Keywords:** DNA damage response, ubiquitin-proteasome system, RNA interference screens, PARP inhibitors, CBLC

## Abstract

Based on a series of basic, preclinical and clinical studies, the Poly (ADP-ribose) Polymerase 1 (PARP1) inhibitor, olaparib, has recently been approved for use in ovarian cancer patients with *BRCA1* or *BRCA2* mutations. By identifying novel predictive biomarkers of tumour cell sensitivity to olaparib, it is possible that the utility of PARP inhibitors could be extended beyond this patient subgroup. Many of the known genetic determinants of PARP inhibitor response have key roles in DNA damage response (DDR) pathways. Although protein ubiquitylation is known to play an important role in regulating the DDR, the exact mechanisms by which this occurs are not fully understood. Using two parallel RNA interference-based screening approaches, we identified the E3 ubiquitin ligase, CBLC, as a candidate biomarker of response to olaparib. We validated this observation by demonstrating that silencing of CBLC causes increased sensitivity to olaparib in breast cancer cell line models and that defective homologous recombination (HR) DNA repair is the likely cause. This data provides an example of how defects in the ubiquitin machinery have the potential to influence the response of tumour cells to PARP inhibitors.

## INTRODUCTION

The poly (ADP-ribose) polymerase, PARP1 plays a key role in the repair of damaged DNA [1]. Upon binding damaged DNA, PARP1 uses β-NAD+ as a co-factor to synthesise poly (ADP-ribose) chains on a series of target proteins [1]. This PARylation of PARP1 substrates initiates the localisation of a series of DNA repair mediators to the site of DNA damage, before autoPARylation of PARP1 causes its release from DNA [1]. Over the past few years, a number of small molecule inhibitors of PARP1 have been developed for the treatment of cancer [2]. Pre-clinical work demonstrated that these inhibitors cause synthetic lethality in tumour cells with *BRCA1* or *BRCA2* gene defects [3, 4], an observation that has also been confirmed in clinical trials [2, 5]. It seems likely that the sensitivity of *BRCA* defective tumour cells to PARP inhibitors is caused by their characteristic defect in repair of DNA double strand breaks (DSBs) by homologous recombination (HR), a process controlled by BRCA1, BRCA2 and the DNA recombinase RAD51 [3]. Although various mechanisms to explain this synthetic lethality have been proposed, one hypothesis is that PARP inhibitors restrict the release of PARP1 from damaged DNA [6]. The DNA lesion that results from the trapping of PARP1 on DNA likely stalls DNA replication forks and requires functional HR for its repair [6, 7].

In addition to defects in *BRCA1* and *BRCA2,* preclinical work has suggested that alterations in a series of additional genes also modulate the response to PARP inhibitors. In totality these efforts have identified a number of candidate predictive biomarkers of tumour cell response including *ATM, ATR, CHEK1, CHEK2, DSS1, RAD51, NBS1*, *IPMK*, *NAMPT, ERCC1,* the Fanconi anaemia complementation genes, *PTEN* and the *TMPRSS2–ERG* and *EWSR1-FLI1* translocations (reviewed in [2]). Recently, we also described a genome-wide RNA interference screen that identified a compendium of novel PARP inhibitor sensitivity-causing genes, including the kinase-coding gene *CDK12* [8].

The majority of PARP inhibitor sensitivity genes identified to date have a known role in DNA double strand break repair (DSBR). In part, the DSB signalling and repair process is controlled by ubiquitylation, the post-translational, covalent attachment of 76 amino-acid ubiquitin chains to target proteins (reviewed in [9,10]). Ubiquitylation is mediated via the coordinated activity of E1 (ubiquitin activating), E2 (ubiquitin-conjugating) and E3 (ubiquitin ligase) enzymes and can be reversed by the activity of de-ubiquitylating enzymes (DUBs) (reviewed in [9]). The role of these enzymes in DSB detection and repair is best exemplified by their involvement in the recruitment of key DSBR proteins to the site of DNA damage. For example, DNA double strand breaks are recognised by the MRN (Mre11–Rad50–NBS1) complex, an event that leads to the activation of signalling kinases that phosphorylate the histone H2AX on chromatin that flanks the site of DNA damage. This in turn enables the recruitment and phosphorylation of the DSBR mediator, MDC1. Once located to a DSB, MDC1 is itself phosphorylated; the phosphorylated amino acids on MDC1 are bound by the E3 ligase RNF8, which initiates a series of ubiquitylation events that ultimately recruit the E3 ligase RNF168. Together the RNF8 and RNF168 ubiquitylation events enable the recruitment and retention of a series of DSBR factors including BRCA1, which also has E3 ligase activity and drives DSBR by HR, and 53BP1, whose activity drives DSBR via an alternative process known as Non Homologous End Joining (reviewed in [9, 10]). In addition to the role of E1, E2 and E3 enzymes in these processes, a recent systematic analysis of DUBs, which remove ubiquitin residues from proteins, has highlighted the role of these enzymes in DSBR and the maintenance of genomic integrity [11].

Given the known role of ubiquitin metabolism in DSBR and the response to PARP inhibitors being determined by this process, we assessed the possibility that additional genes involved in ubiquitin metabolism might alter the tumour cell response to PARP inhibitors. To do this, we have performed a high-content microscopy-based genetic screen for ubiquitylation-related genes, along with parallel analyses of data from a recent genome-wide RNA interference screen and integrated these two complementary datasets in this study. Below, we describe these parallel approaches, as well as functional studies that together pinpoint the ubiquitin ligase CBLC as a previously unrecognized factor whose depletion is synthetically lethal with PARP inhibition.

## RESULTS

### Genome-wide PARPi shRNA screen identifies candidate olaparib sensitisation genes involved in the control of ubiquitylation

To investigate the possibility that proteins involved in the ubiquitylation machinery modulated the tumour cell response to a clinical PARP inhibitor, olaparib [3, 12], we first reanalyzed data from a previously published genome-wide olaparib sensitization genetic screen [8]. In this screen, an olaparib resistant, *BRCA1, BRCA2* and *p53* wild-type breast tumour cell line, MCF7, was transduced with a lentiviral library encompassing 57,540 short hairpin (sh)RNA expression constructs designed to target 16,487 unique human protein-coding genes. The virally transduced cell population was subsequently divided into two cohorts, one exposed to olaparib for two weeks, the other exposed to the drug vehicle. By comparing shRNA frequencies in olaparib *vs.* vehicle surviving cell populations after two weeks, we identified 2,208 different genes whose gene silencing was predicted to result in enhanced olaparib sensitivity (those shRNAs that gave an olaparib sensitization Z score <-2) [8]. By re-annotating the candidate olaparib sensitisation genes identified in this screen using pathway annotation tools such as KEGG, we identified a series of genes implicated in the control of ubiquitylation that were also implicated in olaparib sensitivity (Table 1). This gene list included both E1 Ubl-activating enzymes, E2 Ubl-conjugating enzymes as well as E3 Ubl-protein ligases and included known determinants of PARP inhibitor sensitivity such as *BRCA1* [3] as well as the *UBA1* E1 enzyme coding gene, previously reported to be required for responses to IR and replication stress in human cells (Supplementary Figure 1) [13]. Amongst these olaparib candidate sensitivity genes, we also noted *RNF168*, which is known to play a key role in the recruitment of a series of DSBR factors [9, 10, 14] as well as a series of DUB enzyme-coding genes. These latter genes included *BAP1*, required for histone H2A deubiquitylation [15], *BRCC36 (BRCC3)* which has been associated with the *in vitro* response to the clinical PARP inhibitor rucaparib [16] and *USP7S*, whose gene product is involved in controlling the p53 response to DNA damage [17].

**Table 1:** Genes implicated in Ubl metabolism identified as candidate olaparib sensitivity genes in [8] The extent of olaparib sensitisation is indicated by the median Z score. Negative Z scores indicate a sensitization effect.

Gene Symbol	Median Drug EffectZScore	Gene name
CBLC	−7.67	Cas-13r-M (murine) ecotropic retroviral transforming sequence c
MDM2	−7.46	Mdm2 p53 binding protein homolog (mouse)
ASB5	−5.77	ankyrin repeat and SOCS box containing 5
BRCAI	−4.38	breast *cancer* I, early onset
SOCS6	−4.11	suppressor of cytokine signaling 6
TMEM 189- UBE2V I	−4	TMEM I 89-UBE2V I readthrough
WWP I	−4	WW domain containing E3 ubiquitin protein ligase 1
FBX03	−3.99	F-box protein 3
SUMO I	−3.97	SMT3 suppressor of mif two 3 homolog 1 (S. cerevisiae)
RLIM	−3.92	ring finger protein, LIM domain interacting
RNFI28	−3.64	ring finger protein 128
FBX046	−3.49	F-box protein 46
USP38	−3.43	ubiquitin specific peptidase 38
UBE2C	−3.41	ubiquitin-coniugating enzyme E2C
BAPI	−3.39	BRCAI associated protein-1 (ubiquitin carboxy-terminal hydrolase)
UBAI	−3.35	ubiquitin-like modifier activating enzyme I
SPOPL	−3.2	speckle-type POZ protein-like
FBX0I 1	−3.19	F-box protein 11
UBR7	−3.18	ubiquitin protein ligase E3 component n-recognin 7 (putative)
1:13XW4	−3.14	F-box and WD repeat domain containing 4
RNFI33	−3.09	ring finger protein 133
RNF2	−3.03	ring finger protein 2
ATG4C	−3.01	ATG4 autophagy related 4 homolog C (S. cerevisiae)
P1AS I	−3	protein inhibitor of activated STAT, 1
KLHL2 I	−2.98	kelch-like 21 (Drosophila)
TCEB I	−2.94	transcription elongation factor 13 (SIM, polypeptide 1 (I 5kDa, elongin C)
USP7	−2.94	ubiquitin specific peptidase 7 (herpes virus-associated)
ITCH	−2.93	itchy E3 ubiquitin protein ligase homolog (mouse)
MALTI	−2.93	mucosa associated lymphoid tissue lymphoma translocation *gene* 1
USP I 0	−2.87	ubiquitin specific peptidase 10
ZBTB16	−2.86	zinc finger and BTB domain containing 16
NEURLIB	−2.8	neuralized homolog I B (Drosophila)
SMURF2	−2.79	SMAD specific £3 ubiquitin protein ligase 2
DNAHI2	−2.73	dynein, axonemal, heavy chain 12
XIAP	−2.72	X-linked inhibitor of apoptosis
BARDI	−2.71	BRCAI associated RING domain 1
UBE2R2	−2.71	ubiquitin-conjugating enzyme E2R 2
CUL7	−2.67	cullin 7
RNF144A	−2.65	ring finger protein I 44A
UBE2G2	−2.63	ubiquitin-conjugating enzyme E2G 2 (UBC7 homolog, yeast)
RNF168	−2.62	ring finger protein 168

Gene Symbol	Median Drug EffectZScore	Gene name
RNF41	−2.61	ring finger protein 41
SKP2	−2.6	S-phase kinase-associated protein 2 (p45)
UBE4A	−2.6	ubiquitination factor E4A (UFD2 homolog, yeast)
KLHL36	−2.56	kelch-like 36 (Drosophila)
HECTD4	−2.55	chromosome 12 open reading frame 51
USP8	−2.55	ubiquitin specific peptidase 8
BRCC3	−2.54	BRCA I /BRCA2-containing complex, subunit 3
CDC27	−2.51	cell division cycle 27 homolog (S. cerevisiae)
UBOX5	−2.46	U-box domain containing 5
UFSP2	−2.44	UFM1-specific peptidase 2
ATG4D	−2.42	ATG4 autophagy related 4 homolog D (S. cerevisiae)
WWP2	−2.34	WW domain containing E3 ubiquitin protein lipase 2
SHPRH	−2.33	SNF2 histone linker PHD RING helicase
IS015	−2.3	ISGIS ubiquitin-like modifier
KLHL3	−2.3	ketch-like 3 (Drosophila)
ATG 10	−2.29	ATG 10 autophagy related 10 homolog (S. cerevisiae)
ATG4A	−2.28	ATG4 autophagy related 4 homolog A (S. cerevisiae)
TR1M31	−2.25	tripartite motif containing 31
ATG7	−2.22	ATG7 autophagy related 7 homolog (S. cerevisiae)
USP9Y	−2.22	ubiquitin specific peptidase 9, Y-linked
FBXL5	−2.21	F-box and leucine-rich repeat protein 5
UBE2EI	−2.21	ubiquitin-conjugating enzyme E2E I (UBC4/5 homolog, yeast)
RINGI	−2.19	ring finger protein 1
MYCBP2	−2.16	MYC binding protein 2
ANAPC1	−2.15	anaphase promoting complex subunit I
FBXL13	−2.15	F-box and leucine-rich repeat protein 13
USP33	−2.14	ubiquitin specific peptidase 33
USP42	−2.12	ubiquitin specific peptidase 42
ASB 16	−2.11	ankyrin repeat and SOCS box containing 16
OTUD713	−2.11	OTU domain containing 7B
UEVLD	−2.11	UEV and lactatehnalate dehyrogenase domains
FBX027	−2.1	F-box protein 27
UBE2O	−2.1	ubiquitin-conjugating enzyme E20
ASB7	−2.09	ankyrin repeat and SOCS box containing 7
FBX040	−2.09	F-box protein 40
USP I	−2.09	ubiquitin specific peptidase 1
NPLOC4	−2.08	nuclear protein localization 4 homolog (S. cerevisiae)
IlECTD1	−2.06	HECT domain containing 1
TRIMS	−2.06	tripartite motif containing 5
ANAPC4	−2.04	anaphase promoting complex subunit 4
CANDI	−2.04	cullin-associated and neddylation-dissociated 1
BTBD I	−2.03	BIB (POZ) domain containing 1
FEM IC	−2.03	fem-I homolog c (C. elegans)
LRRC29	−2.03	leucine rich repeat containing 29
USP21	−2.03	ubiquitin specific peptidase 21
FBX038	−2.01	F-box protein 38
DCAF13	−2	DDB1 and CUL4 associated factor 13

Gene Symbol	Median Drug EffectZScore	Gene name
FBXO24	−2	F-box protein 24
FEM IA	−1.98	fern-I homolog a (C. elegans)
FBX039	−1.96	F-box protein 39

**Figure 1:**
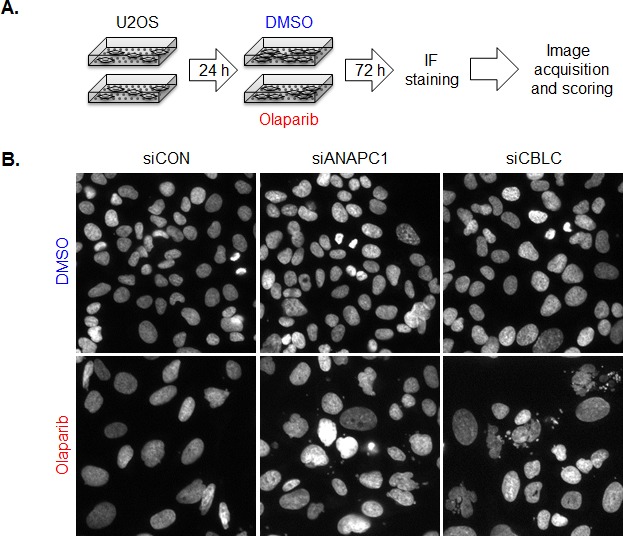
Focused siRNA screen for Ubl modifiers of the PARP inhibitor response A. Screening procedure. U2OS cells were plated on siRNA arrays and 24 h later treated with 10 uM Olaparib or vehicle (DMSO). After 3 days of cultivation cells were fixed, cells nuclei were stained with Hoechst and images corresponding to each siRNAs acquired using automated routine. Scoring was done in two ways; first using automated routine in program ImageJ number of micronuclei was determined. Manual scoring of nuclear and mitotic defects was employed as another way to determine which siRNA knockdowns sensitize cells to Olaparib. B. Images of Hoechst stained cell nuclei from siRNA screen illustrating the effect of siRNA targeting CBLC.

### A parallel high-content microscopy-based genetic screen identifies ubiquitylation-related determinants of PARP inhibitor sensitivity

In parallel to the genome-wide genetic screen, we also carried out a focused high-content genetic screen to identify genes associated with ubiquitin metabolism that are synthetically lethal with PARP inhibition. In this screen, the human U2OS osteosarcoma-derived cell line was plated on an siRNA array designed to target 1346 human genes involved in the ubiquitin-metabolism, the proteasome system and genes encoding zinc-finger proteins [13]. Following siRNA transfection, cells were exposed to either olaparib or the drug vehicle, DMSO for three consecutive days (Figure 1A). At this point, cells were fixed, nuclei stained with Hoechst and images of cell nuclei were automatically acquired using wide-field fluorescence microscope. We estimated the effect of each siRNA on olaparib sensitivity and genomic instability using two approaches. First, we counted micronuclei formation as an estimate of genomic instability caused by PARP inhibitor exposure using an automated ImageJ-based programme, and compared micronuclei frequency in olaparib and vehicle exposed cells (Table 2). In parallel, we also used manual scoring of nuclear and mitotic defects (multinucleation, irregularity of nuclear shape and anaphase chromosome bridges) (Table 3).

**Table 2:** Genes implicated in olaparib sensitivity as scored by automated micronuleation analysis Hits are defined as those that showed more than 31 MN in the presence of Olaparib and 15 or less MN in DMSO.

MICRONUCLEATION HITS				
4 of 4 (3)	3 of 4 (14)	2 of 4 (104)		
CUL7	ANAPC1	ANAPC2	HGS	RNF149
MAP3K7IP2	ASB6	ANAPC7	HIC1	RNF182
MYO1E	ATR	ANKRD13A	HLTF	RNF187
	DHX9	ASB11	JARID1A	RNF19A
	ERLIN1	ASB14	KALRN	RWDD2A
	MDM1	ASB15	KBTBD6	SENP2
	POLK	ASB17	KBTBD7	SENP5
	RNF17	BAG1	KIAA0999	SENP8
	RNF4	BCL6B	KIAA1333	SHPRH
	STAMBPL1	BIRC2	LNX2	SMURF2
	UBE2H	BMI1	LRPPRC	SQSTM1
	UBQLN1	BTBD14A	MARCH10	STUB1
	ZBTB3	CAND2	MARK2	SUGT1
	ZNF395	CBLL1	MKRN2	TBK1
		CCNF	MLL2	TEX13A
		CDH1	NOSIP	TMEM183A
		COPS3	NSD1	TOM1
		COPS5	NSFL1C	TOPBP1
		CSMD3	NSMCE2	TRAF3
		DDB2	OTUD5	TRAF5
		DERL2	OTUD7A	TREX2
		DIP2C	PARD6B	TRIM23
		DSG1	PHF12	TRIM8
		DTX4	PHF21A	TTC3
		EIF2AK4	PHF7	UBL3
		EPN3	POLI	UBL4B
		FAM100A	PSMA5	UBXD5
		FAU	PSMA8	USP45
		FBXO34	PSMF1	VSP13A
		FBXW11	PYGO2	WDR24
		FZR1	RB1CC1	WDR32
		G3BP1	RCBTB1	XAF1
		GZF1	RFPL2	ZBTB17
		HADHA	RFWD2	ZNFX1
		HECTD1	RNF139	

**Table 3:** Genes implicated in olaparib sensitivity by manual scoring of nuclear and mitotic defects

MANUAL SCORING HITS		
4 of 4 (0)	3 of 4 (2)	2 of 4 (21)
	ATR	ANUBL1
	FBOX5	ARIH1
		AURKA
		CBLC
		CCNF
		CDC20
		CDC27
		CUL1
		DDB1
		DDB2
		DHX9
		DPF2
		FBXW7
		PEX10
		PHF21A
		PIAS4
		RNF17
		RNF4
		SKP1
		SPSB2
		UBQLN3

In both sets of analyses, we used our previously established high-content microscopy-based screening strategy [13,14] and assessed the phenotypes in four independent transfections, imaging approximately 150 cells that usually occupy each individual siRNA spot. Across the entire screen, our automated analysis examined the average number of micronuclei for each of the computer-identified multicellular siRNA spots. In control DMSO-exposed cells this overall micronuclei score was 2.63, while in control olaparib-exposed cells, this overall average score was 15.35 (baseline average score). We considered as “hits” those siRNAs that elicited a greater than two-fold increase (30.7 or more micronuclei/spot) in the frequency of micronuclei when exposed to olaparib, but did not cause an alteration in micronuclei frequency in DMSO exposed cells. Using this approach, the automated imaging analysis identified three genes (*CUL7, MAP3K7IP2, MYO1E*) that enhanced olaparib-induced micronucleation in all four transfections, 14 genes with three out of four transfections scoring positively, and 104 genes where two out of four transfections elicited micronucleation in response to PARP inhibitor exposure (Table 2). An example of the PARP inhibitor induced micronucleation phenotype is shown in Figure 1B, where the effect of the siRNA targeting *ANAPC1 (*anaphase promoting complex subunit 1), an E3 ubiquitin ligase coding gene is shown. We noted that *ANAPC1* also scored in the genome-wide shRNA olaparib sensitivity screen as a determinant of olaparib sensitvity (DE Z score of −2.15, Table 1).

Using manual scoring, no single gene scored in all four transfections but we identified two genes where siRNA caused aberrations in the examined nuclear parameters in the olaparib exposed cells in three out of four transfections; the known olaparib sensitivity gene *ATR* (ATR serine/threonine kinase) and *FBXO5* (F-box protein 5, Table 3) a new olaparib-sensitizing factor identified in our present screen. siRNA targeting 21 genes caused nuclear defects in the olaparib exposed cells in two out of four transfections (Table 3). Of these genes, we noted that the siRNA designed to target the E3 ligase coding gene *CBLC (*Cbl proto-oncogene C [18]) caused pronounced nuclear aberrations in olaparib-exposed cells but not in control DMSO-exposed cells (Figure 1B). shRNA designed to target *CBLC* also caused one of the most profound olaparib sensitisation effects in the MCF7 genome-wide shRNA interference screen (Z score −7.67, Table 1). By comparison, shRNA targeting BRCA1 gave an olaparib drug sensitisation Z score of −4.38.

### CBLC silencing causes a HR defect

**Figure 2:**
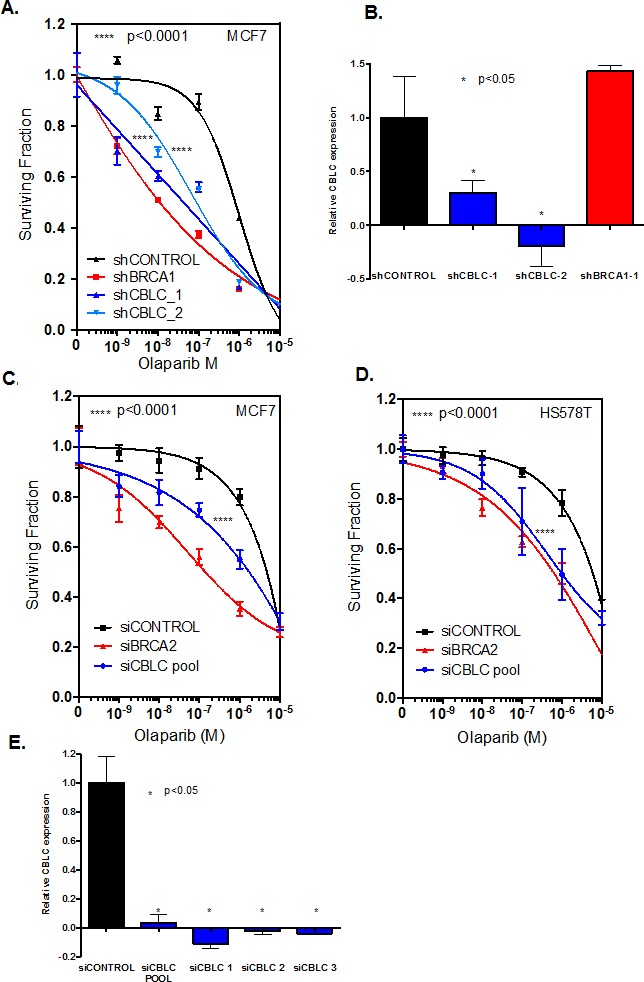
CBLC gene silencing causes PARP inhibitor sensitivity A. Clonogenic dose-response survival curves from MCF7 human breast cancer cells infected with shRNA expression constructs targeting either *BRCA1* or *CBLC*. Transduced cells were subsequently exposed to olaparib for 14 days at which point cell colonies were counted. Two different shRNA expression constructs targeting CBLC, shCBLC_1 and shCBLC_2, were used.****ANOVA *p* value <0.0001 for the dose response curves in either shCBLC_1 or shCBLC_2 transduced cells *vs.* shCONTROL transduced cells. Data from shBRCA1 transduced cells is shown as the positive control. B. Bar chart illustrating CBLC RT-PCR data from MCF7 cells expressing CBLC cDNA and transduced with shRNA expression constructs. *Student's *t* test *p* value <0.05 for CBLC expression compared to shCONTROL tranduced cells. C. and D. Olaparib dose-response survival curves from MCF7 (C) or HS578T (D) human breast cancer cells transfected with siRNA targeting either *BRCA2* or *CBLC*. Cells were transfected with siRNA and 48 hours later exposed to olaparib for a subsequent six days.****ANOVA *p* value <0.0001 for the dose response curves in siCBLC transfected cells *vs.* siCONTROL transfected cells. Data from siBRCA2 transfected cells is shown as the positive control. E. Bar chart illustrating CBLC RT-PCR data from MCF7 cells expressing CBLC cDNA and transfected with siRNA. * Student's *t* test *p* value <0.05 for CBLC expression compared to siCONTROL transfected cells. Where error bars are shown these represent the standard error of the mean (SEM) from three independent experiments.

Given the profound effect of shRNA targeting *CBLC* in the genome-wide genetic screen in MCF7 cells and the effect of siRNA targeting *CBLC* on olaparib-induced nuclear defects in U20S cells, we assessed the extent of PARP inhibitor sensitivity caused by CBLC silencing in dose-response clonogenic survival experiments. When compared to a control, non-targeting shRNA expression construct, two different CBLC shRNA expression constructs caused a profound and significant increase in olaparib sensitivity in MCF7 cells (Figure 2A, olaparib survival in shCONTROL targeted cells vs. shCBLC targeted cells, p<0.0001 ANOVA, and Figure 2B). We found the extent of olaparib sensitivity caused by CBLC shRNA to be equivalent to that caused by an shRNA expression construct designed to target BRCA1 (Figure 2A). We also found that siRNA reagents designed to target CBLC caused olaparib sensitisation in MCF7 cells as well as in a second breast tumour cell line model, HS578T (Figure 2C,D,E), suggesting that these effects were neither restricted to the method of RNA interference used nor to the MCF7 cell line model used in the original genome wide shRNA screen.

**Figure 3:**
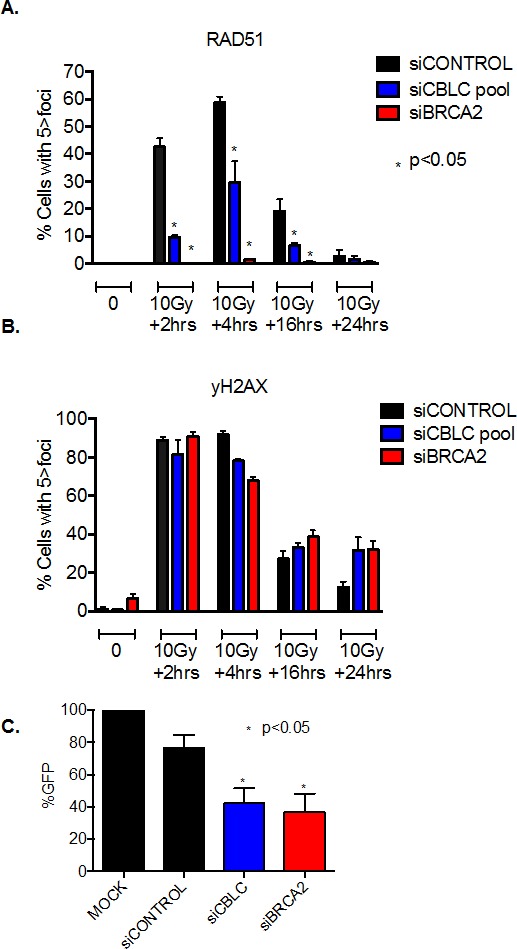
CBLC gene silencing causes a homologous recombination defect A and B. Bar charts indicating the frequency of cells with greater than five RAD51 (A) and γH2AX (B) nuclear foci in response to exposure to 10 Gy ionising radiation and CBLC siRNA. CBLC siRNA reduced the frequency of cells with RAD51 foci * p<0.05 Student's *t* test vs. control siRNA transfected cells at the same time point. Data from siBRCA2 transfected cells is shown as a positive control. C. Bar chart indicating GFP signal generated by repair of the DR-GFP DNA substrate. Data shown is normalised to the % of GFP positive cells in a mock-transfected sample. CBLC siRNA reduced the frequency of GFP+ve cells *p<0.05 Student's *t* test vs. control siRNA transfected cells. Data from siBRCA2 transfected cells is shown as a positive control. Where error bars are shown these represent the standard error of the mean (SEM) from three independent experiments.

One key molecular determinant of tumour cell response to PARP inhibition is the ability to localise the DNA recombinase RAD51 to the site of DNA damage, a critical event in HR repair that can be monitored by the immunodetection of DNA damage induced RAD51 foci [3, 19]. We assessed the RAD51 response in human tumour cells transfected with *CBLC* siRNA, as well as the formation of γH2AX foci, a marker of histone H2AX phosphorylation that is associated with DNA DSB formation [20]. Whilst control-transfected MCF7 cells exhibited a clear increase in both RAD51 and γH2AX foci in response to ionising radiation (IR) (Figure 3A and B), cells transfected with either *CBLC* or *BRCA2* siRNA exhibited a clear reduction in the frequency of cells exhibiting a RAD51 foci response (Figure 3A and B). In addition to the RAD51 defect, we also noted that 24 hours after the initial exposure to IR, the frequency of cells still exhibiting γH2AX foci, and presumably unrepaired DSBs, was moderately increased in cells transfected with siRNA targeting either *CBLC* or *BRCA2* (Figure 3B), compared to those transfected with a control siRNA.

Taken together, these results suggested that a *CBLC* defect could cause a reduction in the ability to repair DNA by homologous recombination (HR). To directly assess the effect of CBLC silencing on HR, we estimated HR activity using a synthetic DNA HR substrate, DR-GFP, which generates a GFP signal once an experimentally induced DSB has been repaired by HR [21]. We found that the repair of the DR-GFP substrate by HR was impaired in both *BRCA2* and *CBLC* siRNA transfected cells compared to control transfected cells (Figure 3C, p<0.05 by student's t-test), supporting the hypothesis that loss of CBLC activity causes a HR defect, a phenotype that could explain the PARP inhibitor sensitivity also caused by loss of CBLC function. We also assessed the effect on progression of the MCF7 breast cancer cells through the cell cycle in response to CBLC silencing. In both the presence and absence of IR exposure, we found that CBLC siRNA caused a modest reduction in the relative frequency of cells in both S and G2 phases of the cell cycle (Supplementary Figure 2A and B). These alterations were similar to those elicited by siRNA targeting BRCA2 (Supplementary Figure 2A and B).

Based on these analyses, we conclude that depletion of CLBC sensitizes diverse human cancer cells to the PARP inhibitor olaparib and causes defects in cellular responses to DNA double strand breaks including Rad51 foci formation and HR repair. Furthermore, the extent of the observed phenotypes after CBLC depletion was comparable to depletion of BRCA2, one of the key HR factors currently being used to direct the clinical use of PARP inhibitors.

## DISCUSSION

In the work described here, we used a reanalysis of a genome-wide genetic screen [8] together with a focused siRNA screen and identified and validated a novel genetic determinant of tumour cell PARP inhibitor sensitivity, the ubiquitin ligase CBLC [18]. siRNA targeting CBLC enhanced the extent of nuclear defects monitored as a readout in the focused screen in cells exposed to the clinical PARP inhibitor olaparib and impaired DNA damage-induced RAD51 foci formation. This suggested that the cause of PARP inhibitor sensitivity in cells depleted of CBLC might be defective homologous recombination. This hypothesis was also supported by our experiments using a synthetic HR DNA substrate, the results of which were consistent with the concept of homologous recombination being indeed defective when CBLC is silenced.

*CBLC* encodes an E3 ubiquitin ligase of the CBL family, whose other members include CBL (also known as c-CBL) and CBLB [18]. Like CBL and CBLB, CBLC has a highly conserved N-terminus that encompasses a phosphotyrosine binding domain and a catalytic ring finger domain [22, 23]. Compared to CBL and CBLB, relatively little is known about the function of CBLC. Similar to CBL and CBLB, CBLC binds SH3 (SRC Homology 3) domain containing proto-oncogenic tyrosine kinases such as EGFR (Epidermal Growth Factor Receptor), LYN, CRK, SRC and RET [22, 24, 25]. EGF-triggered activation of EGFR results in CBLC being recruited to the receptor where CBLC is phosphorylated. The interaction between CBLC and EGFR leads to the attenuation of MAP kinase signalling [22]. Likewise, CBLC enhances ubiquitylation and degradation of the oncoprotein RET [26, 27]. CBLC has also been shown to form a complex with HIC5 (Hydrogen peroxide-inducible clone 5) and the heat shock protein HSP27. These latter interactions are thought to be required for the ubiquitylation of NOX4, a protein involved in the control of reactive oxygen species [28] and diverse stress-induced cellular responses including oncogene-induced cell senescence [29]. It remains to be seen whether any of the known interactions with CBLC are responsible for the PARP inhibitor sensitivity phenotype we have observed here or whether another, as yet unidentified, CBLC substrate mediates these effects.

As far as we are aware, this is the first report to link CBLC to a DNA repair related function. Compared to other genes implicated in HR and PARP inhibitor sensitivity, *CBLC* mutations are relatively rare in cancer, being present in approximately 2 % of 4000 human tumours whose exome sequence is described on the cBio database [30, 31] This of course does not exclude the possibility that impaired *CBLC* transcription, translation or reduction in CBLC protein levels by enhanced turnover could cause sensitivity to a PARP inhibitor. Unlike its relatives within the CBL family, CBLC expression appears to be restricted to epithelial tissues [32,33]. Transgenic expression of CBLC in a mouse mammary gland causes impairment of cell proliferation, suggesting that CBLC might have a growth-inhibitory role [32]. The role that CBLC plays in DNA repair by homologous recombination, described here, might suggest that this ubiquitin ligase could represent a candidate for a novel tumour suppressor. It is therefore plausible that transcriptional, translational or proteolytic suppression of CBLC in tumours might be a more common event than gene mutations. Furthermore, in contrast to defects in genes such as *BRCA1* [34], constitutive *Cblc* deficiency is neither embryonically lethal in mice nor overtly harmful in somatic cells of adult *Cblc* deficient animals [33].

Given the recent regulatory approval in the USA and Europe for olaparib (now named Lynparza) as a treatment for BRCA1/2-deficient ovarian tumours [5], the functional assessment and validation of candidate biomarkers is most timely. Apart from the emerging sensitivity biomarkers identified through synthetic lethal interactions with PARP inhibitors [1, 2,7,8,12,34-38], several molecular determinants of enhanced resistance in BRCA-deficient tumours, such as revertant *BRCA* gene mutations, loss of p53BP1 or JMJD1C have been reported and their predictive value requires careful evaluation [2,34,39-41]. Similarly, it remains to be seen whether CBLC modulates the clinical response to PARP inhibitors, but the work described here suggests that along with other genes that are known to control homologous recombination, CBLC should be considered as a candidate biomarker of response and potentially assessed in biopsy material from patients enrolled on existing and future PARP inhibitor clinical trials [2].

## MATERIALS AND METHODS

### Cell lines

Human U2OS cell line was cultured in Dulbecco's Modified Eagle's medium (DMEM) supplemented with 10% (v/v) foetal bovine serum (Invitrogen, Carlsbad, CA, USA) and penicillin/streptomycin (Sigma-Aldrich, St. Louis, MO, USA). Human MCF7 and Hs578T cell lines were grown in RPMI 1640 or DMEM supplemented with 2mM glutamine, 10% (v/v) foetal bovine serum and penicillin/streptomycin respectively.

### Chemicals

PARP inhibitor olaparib was purchased from Selleckchem and was dissolved in DMSO (Sigma-Aldrich, St. Louis, MO, USA).

### shRNA screen analysis

Median drug effect Z score data from [8] was used. Genes with median drug effect Z scores of <-2 in [8] were cross referenced with gene listed in the following KEGG groups to generate the data shown in Table 1: Ubiquitins and Ubiquitin-like proteins, Ubiquitin-activating enzymes (E1), Ubiquitin-conjugating enzymes (E2), Ubiquitin ligases (E3), Deubiquitinating enzyme (DUB).

### Targeted ubiquitin-proteasome siRNA-based screen

A custom-designed siRNA arrays were described previously [13]. U2OS cells were plated on siRNA arrays and 24 h later exposed to 10 μM Olaparib or DMSO. Olaparib treated arrays were prepared in duplicate. After 72 h of cultivation cells were fixed and stained with Hoechst (Invitrogen, Carlsbad, CA, USA). Image acquisition of the siRNA arrays was described previously [13]. The number of micronuclei was determined by automated routine using software ImageJ (http://rsb.info.nih.gov/ij/). In addition to automatic scoring of micronuclei induction, manual scoring based on other nuclear and mitotic defects (multinucleation, irregularity of nuclear shape, anaphase bridges) was employed. Hits were defined based on number of micronuclei (MN) in Olaparib control siRNA (siCON + Olaparib) treated cells, where average number of MN per siRNA spot in whole screen was 15.35. Threshold for hits in Olaparib treated cells was set as twice that number (2 × 15.35 = 30.7). As hits were considered siRNAs with more than 31 MN in the presence of Olaparib and 15 or less MN in DMSO control.

### HR assay

A synthetic repair reporter was used as previously described [8]. HeLa cells harboring a single-copy genomic integration of the DR-GFP reporter were transfected with siRNA targeting CBLC, BRCA2 or a siControl. 24 hours later, cells were transfected with the I-*Sce*I expression vector, pcBASce [21]. Forty-eight hours later, HR frequency was estimated by quantifying the frequency of GFP positive cells using FACS [21].

### Immunocytochemistry

The quantification of nuclear RAD51 foci was performed as previously described [3]. Briefly, MCF7 cells transfected with siRNA targeting CBLC, BRCA2 or siCONTROL were plated onto cover slips (BD Biosciences, Oxford, UK). 16 hours later, cells were exposed to 10 Gy ionizing radiation. At 0, 2, 4, 16 and 2 hours after damage, cells were fixed, permeabilized, then immunostained with primary antibody targeting RAD51 (Santa Cruz Biotech) or phospho-yH2AX (Millipore) and detected with a Texas red conjugated secondary antibody. DAPI staining was used to detect nuclei. Nuclear foci were visualized by confocal microscopy and a minimum of 100 fields of view were assessed. Each experiment was repeated twice.

### Cell-based assays

Cell lines were transfected with SMARTpool siRNAs (Dharmacon, GE Healthcare) targeting BRCA2 and CBLC or siCONTROL A (Santa Cruz Biotech) using RNAiMax (Invitrogen) transfection reagent. Cell lines were infected with GIPZ shRNA constructs packaged as lentivirus and after 72hrs selected with 2μg/ml puromycin.

Clonogenic survival assays were performed as previously described [3]. Short-term survival assays were performed in 96-well plates. Cells were seeded in 96-well plates and drug was added after 24 hours. Cell viability was estimated after seven days using Cell-Titre Glo (Promega). Surviving fractions (SFs) were calculated and drug sensitivity curves plotted as previously described [3].

### Quantitative RT-PCR

Cells were transfected with CBLC cDNA expression pCMV6 construct (Origene) then 24 hours later either infected with CBLC or control shRNA or transfected with CBLC or control siRNA. Quantitative RT-PCR was carried out using Assay-on-Demand primer/probe sets (Applied Biosystems). Gene expression was calculated relative to the expression of *GAPDH*, and adjusted relative to expression in shCONTROL or siCONTROL infected cells.

## SUPPLEMENTARY MATERIAL AND FIGURES


