# Supplementary material for: Complementary genetic screens identify the E3 ubiquitin ligase CBLC, as a modifier of PARP inhibitor sensitivity
Source: Oncotarget. 2015 Mar 18;6(13):10746–58. doi: 10.18632/oncotarget.3628 (PMC4484416; doi:10.18632/oncotarget.3628)
Supplement: Supplementary file 1 [file oncotarget-06-10746-s001.pdf]

Complementary genetic screens identify the E3 ubiquitin ligase CBLC, as a modifier of PARP inhibitor sensitivity

Supplementary Material

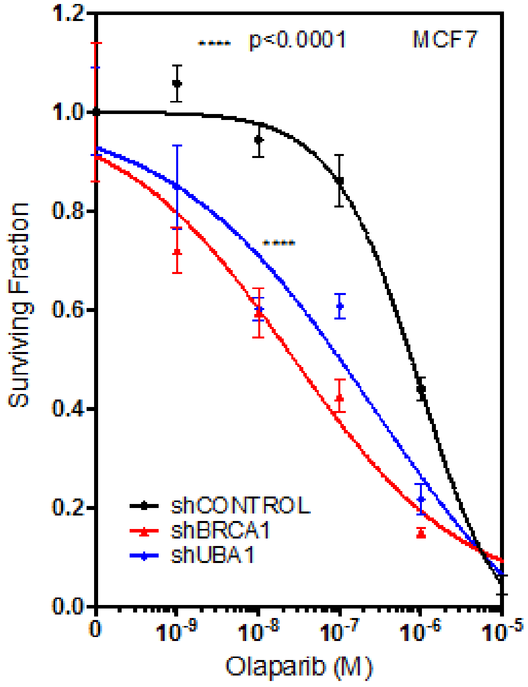

Supplementary Figure 1: CBLC gene silencing causes PARP inhibitor sensitivity. Clonogenic dose-response survival curves are shown from MCF7 human breast cancer cells infected with viral shRNA expression constructs targeting either *BRCA1* or *UBA1*. shRNA transduced cells were exposed to olaparib for 14 days at which point cell colonies were counted. shRNA expression constructs targeting UBA1 were used.****ANOVA p value <0.0001 for the dose response curves in shUBA1 transduced cells *vs.* shCONTROL transduced cells. Data from shBRCA1 transduced cells is shown as the positive control.

A

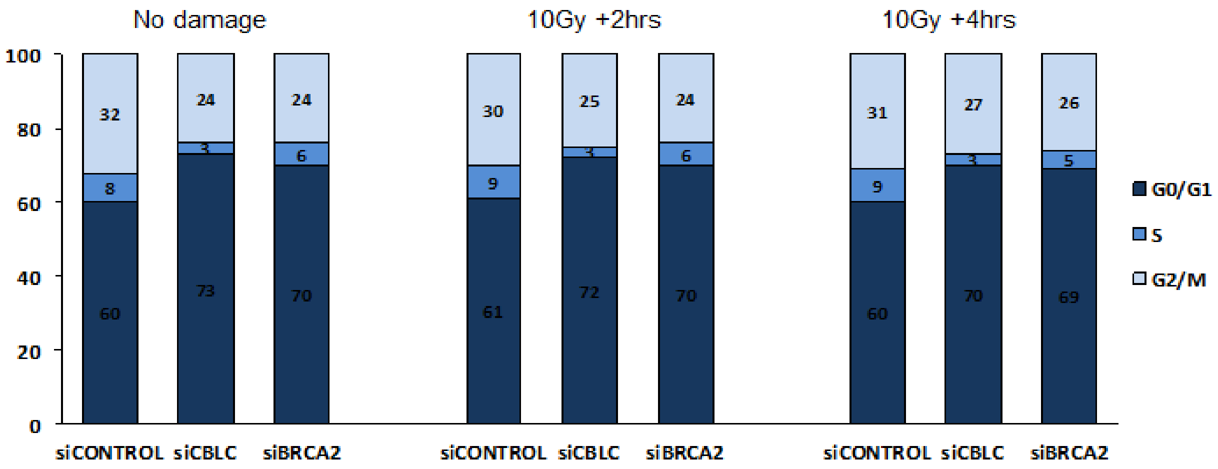
No damage
10Gy +2hrs
10Gy +4hrs
100
80
60
40
20
0
32
8
60
24
3
73
24
6
70
30
9
61
25
3
72
24
6
70
31
9
60
27
3
70
26
5
69
siCONTROL
siCBLC
siBRCA2
siCONTROL
siCBLC
siBRCA2
siCONTROL
siCBLC
siBRCA2
G0/G1
S
G2/M

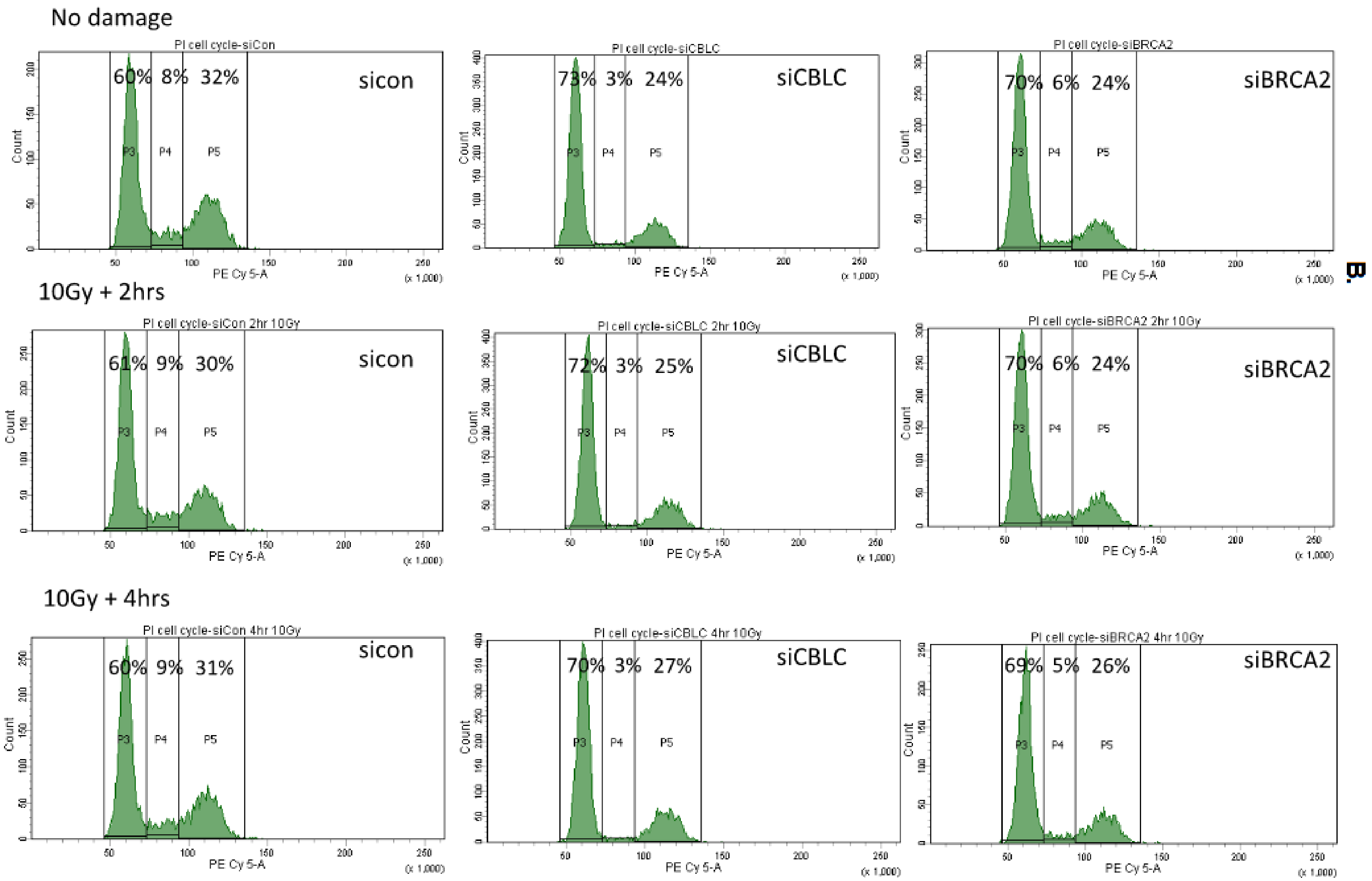
B.
No damage
PI cell cycle-siCon
60% 8% 32%
sicon
PI cell cycle-siCBLC
73% 3% 24%
siCBLC
PI cell cycle-siBRCA2
70% 6% 24%
siBRCA2
10Gy + 2hrs
PI cell cycle-siCon 2hr 10Gy
61% 9% 30%
sicon
PI cell cycle-siCBLC 2hr 10Gy
72% 3% 25%
siCBLC
PI cell cycle-siBRCA2 2hr 10Gy
70% 6% 24%
siBRCA2
10Gy + 4hrs
PI cell cycle-siCon 4hr 10Gy
60% 9% 31%
sicon
PI cell cycle-siCBLC 4hr 10Gy
70% 3% 27%
siCBLC
PI cell cycle-siBRCA2 4hr 10Gy
69% 5% 26%
siBRCA2
P3
P4
P5
Count
PE Cy 5-A
(x 1,000)

Supplementary Figure 2: A and B Effect of CBLC silencing on the cell cycle profile. FACScan plots from irradiated MCF7 cells transfected with control siRNA and siRNA targeting CBLC or BRCA2 are shown. Cells were fixed at time points before and after irradiation and the cell cycle profile was analysed by FACS after PI staining.
